# *Pueraria lobata*–*Prunus mume* Complex Alleviates Alcoholic Liver Disease by Regulating Lipid Metabolism and Inhibiting Inflammation: A Transcriptome and Gut Microbiota Analysis

**DOI:** 10.3390/foods13152431

**Published:** 2024-08-01

**Authors:** Ruixi Gao, Qi Huang, Yanfeng Zeng, Dandan Chen, Ziming Jia, Bingchen Han, Xianju Huang, Qiang Wang, Xin Hu, Maochuan Liao, Jun Li

**Affiliations:** 1 School of Pharmaceutical Sciences, South-Central Minzu University, Wuhan 430074, China; 2023021@mail.scuec.edu.cn (R.G.); 2020110427@mai.scuec.edu.cn (Q.H.); 2022110479@mail.scuec.edu.cn (Y.Z.); 2021110510@mail.scuec.edu.cn (D.C.); xianju@mail.scuec.edu.cn (X.H.); wq@mail.scuec.edu.cn (Q.W.); 2011020@mail.scuec.edu.cn (X.H.); 3030707@mail.scuec.edu.cn (M.L.); 2 Center for Disease Control and Prevention (Hubei Province), Wuhan 430079, China; 3 College of Life Sciences, South-Central Minzu University, Wuhan 430074, China; 2023010071@mail.scuec.edu.cn; 4 Science and Technology Cooperation Base for Evaluation and Utilization of Traditional Medical Resources, South-Central Minzu University, Wuhan 430074, China

**Keywords:** alcoholic liver disease, *Pueraria lobata*–*Prunus mume* complex, transcriptome and gut microbiota analysis, lipid metabolism, anti-inflammation

## Abstract

Background: Lipid metabolism disorder appears to be one of the early features of alcoholic liver disease (ALD), which can be speculated via omics analysis including liver transcriptomics and gut microbiota. A complex consisting of the roots of *Pueraria lobata* and dried fruits of *Prunus mume* (PPC), which possesses hepatoprotective effects, could serve as a drug or functional food. The lack of non-polysaccharide compounds in PPC with their moderation effects on gut microbiota suggests the necessity for a relevant study. Methods: Six groups of Kunming mice (control, Baijiu injury, silybin, low, medium, and high) were modelled by gavage with Baijiu (for 14 days) and PPC (equivalent to a maximum dose of 9 g/kg in humans). The liver transcriptome data were analyzed to predict gene annotation, followed by the verification of gut microbiota, serum, tissue staining, immunohistochemistry, and Western blotting. Liquid chromatography-mass spectrometry was used to detect the components. Results: PPC normalized serum ALT (40 U/L), down-regulated TLR4-NF-κB signaling pathway to inhibit the release of TNF-α (90 pg/mL), improved the expression of occludin, claudin-4, and ZO-1, and restored the abundance of *Muribaculaceae*, *Bacteroides* and *Streptococcus*. Conclusion: PPC can alleviate ALD by regulating the gut microbiota with an anti-inflammatory and intestinal barrier, and has an application value in developing functional foods.

## 1. Introduction

Alcoholic liver disease (ALD) is a common and chronic liver disorder caused by long-term excessive alcohol consumption. The phenotypes of ALD include fatty liver, alcoholic hepatitis, liver fibrosis, cirrhosis, and cancer, resulting in a severe threat to public health [1]. Although the pathogenesis of ALD has not been completely elucidated, hepatic oxidative stress and inflammation, neutrophil infiltration, dysregulation of amino acid, carbohydrate, and lipid metabolism, gut microbiota dysbiosis, and ferroptosis have been implicated [2]. Among these factors, steatosis appears to be the most common hepatic alteration induced by alcohol intake. On the one hand, alcohol exposure accelerates fatty acid synthesis by downregulating adenosine 5-monophosphate-activated protein kinase, inhibiting the activity of the rate-limiting enzyme acetyl-CoA carboxylase, reducing the levels of the fatty acid synthesis precursor malonyl-CoA, inactivating peroxisome proliferator-activated receptor alpha, and promoting fat accumulation, leading to hepatic oxidative stress and cell death [3]. On the other hand, the deficiency of proteins that regulate lipid peroxidation (e.g., fibronectin type III domain-containing protein 3 B) and abnormal mRNA expression (miR-192-5P) have been demonstrated to increase 4-hydroxynonenal (4-HNE) and malondialdehyde (MDA) content, thereby affecting lipid accumulation in ALD [4]. Commonly used health foods or medicines, such as polysaccharides, flavonoids, and triterpenoids in natural products, have been reported to alleviate ALD because of their diverse hepatoprotective effects [5,6,7]. *Pueraria lobata* and *Prunus mume* can be used as foods or drugs. Modern pharmacological studies have demonstrated that the isoflavonoids and triterpenoids in *P. lobata* and *P. mume* can protect the liver from steatosis and inflammation [8] and regulate lipid accumulation and apoptosis [9,10]. Therefore, both plants have the potential to be developed into hepatoprotective functional foods.

Although previous studies reported that each plant in *P. lobata* or *P. mume* can alleviate ALD, little research has investigated their combined effects. Since the complex of *P. lobata* or *P. mume* (PPC) may be developed as a potential functional food with liver protective activity, it is necessary to conduct omics analysis on the non-polysaccharide bioactive compounds (such as flavonoids or triterpenes) that could alleviate ALD. In this study, we first analyzed the characteristics of the livers of mice with ALD using transcriptomics data, identified the main components in PPC, and attempted to validate the representative changes associated with liver function recovery in mice with ALD after PPC administration. Subsequently, enzyme linked immunosorbent assay (ELISA), immunohistochemistry, and Western blotting (WB) were performed to confirm the reliability of the omics results.

## 2. Materials and Methods

### 2.1. Sample Preparation and Liquid Chromatography–Mass Spectrometry (LC-MS)

The roots of *P. lobata* and dried fruits of *P. mume* were used in this study. The mixed powder of the two materials (1:1, *m*/*m*) was immersed in water for 30 min and then refluxed with hot water three times for 30 min each. Subsequently, the extract solution was concentrated by a rotating evaporator (RE2000A, Yarong Instrument, Shanghai, China) until it was totally dried under reduced pressure by a vacuum drying oven (XMTD-8222, Jinghong Experimental Equipment Company, Shanghai, China) to yield the solid PPC. The overall yield from the initial raw materials to the final solid mixture was 20%. The extract was dissolved in methanol (20 mg/mL), filtered through a 0.22 μm membrane filter (Jinteng Company, Tianjing, China), and analyzed by LC-MS.

Compounds from PPC were analyzed using an ultra-high performance liquid chromatography-mass spectrometry (UPLC-MS/MS) system consisting of a Thermo Q Extractive Plus quadrupole electrostatic field orbital trap mass spectrometer operating in the positive/negative electrospray ionization mode, and an Ultimate 3000 UHPLC system (Thermo Fisher Scientific, Waltham, MA, USA) was used for the separation (equipped with a photo-diode array PDA, detector). The following MS parameters were used: mass range, 80–1000 Da; capillary temperature, 300 °C; ion spray voltage, 3200 V; sheath gas, 40 Arb; aux gas, 10 Arb; and max spray current, 100 µA. A Thermo Accucore AQ C18 chromatographic column (2.1 mm × 100 mm, 2.6 μm; Thermo Fisher Scientific) was used. Using mobile phases consisting of methanol (A) and 0.1% formic acid in water (B), gradient elution was performed as follows: 0–3 min, 5–10% A; 3–7 min, 10–17% A; 7–9 min, 17–20% A; 9–11 min, 20–24% A; 11–14 min, 20–24% A; 14–16 min, 24–28% A; 16–19 min, 28–30% A; 19–21 min, 30–35% A; 21–29 min, 35–37% A; 29–33 min, 37–55% A; 33–38 min, 55–63% A; and 38–45 min, 63–100% A. The column temperature, injection volume, and flow rate were 25 °C, 1 μL, and 0.2 mL/min, respectively.

### 2.2. Animals and Model Establishment

Six-week-old male Kunming (KM) mice (20 ± 2 g) were purchased from the Center for Animal Disease Prevention and Control, Hubei province. The production license number of experimental animals is SCXK (E) 2020-0018, the animal product quarantine certificate number is 42000600047709, and the use license number of experimental units is SCXK (E) 2021-0089. The animals were housed in the SPF animal room of the experimental animal center of South-Central Minzu University following the regulations of the animal ethics committee of the university and the National Institutes of Health guidelines (No: 2019010S; 28 February 2019). The feeding room was maintained at 25 ± 2 °C and 60% ± 10% humidity.

A total of 60 KM mice were randomly divided into 6 groups of 10 mice each after 1 week of adaptive feeding as follows: control group (Con), alcoholic liver injury model group (Mod, feeding with Chinese Baijiu, 10 mL/kg, 30% alcohol content, *v*/*v*), silymarin group (Pos, Aladdin Company, Shanghai, China, administered at a dosage of 27.3 mg/kg according to the drug instructions), low-dose group (Low, 16.26 mg/kg), medium-dose group (Med, 48.78 mg/kg), and high-dose group (Hig, 146.34 mg/kg). Based on mouse–human dose conversion [11] and the extraction rate (20%), the aforementioned doses are equivalent to 1, 3, and 9 g/kg, respectively, in humans. Mice in the Low, Med, and Hig groups were administered the extract via oral gavage once a day for 4 weeks (days 1–28). Mice in the Con and Mod groups received an equal amount of physiological saline. Starting from the third week (day 15), excluding the Con group, each mouse was orally administered Baijiu 4 h after drug administration for the next 2 weeks (days 15–28). On day 28, mouse feces were collected and frozen at −80 °C for 16S rRNA analysis. All mice were fasted (excluding water) for 12 h before the end of the experiment. After day 28, the mice were sacrificed via CO_2_ inhalation, and blood samples were collected from their eyeballs. Liver and ileum tissues were harvested and weighed. A part of the liver and ileum tissues was fixed in 4% paraformaldehyde, and the remaining tissue was stored in a −80 °C freezer. Liver injury and ileum parameters were analyzed by WB, immunohistochemistry, and biochemical analysis. All animals received humane care.

### 2.3. Transcriptome and 16S rRNA Analysis

The raw transcriptomic data of the mouse liver tissue were downloaded from the Gene Expression Omnibus (GEO) database (GSE179398). pyDESeq2 (version 0.4.8) software was used to perform differential analysis. The screening requirements for differentially expressed genes (DEGs) were |log2 (fold change)| > 1.5 and *p* < 0.05. Clusterprofiler (version 4.10.1) was used to compare the DEGs between normal mice and mice with ALD based on the biological process (BP), cellular component (CC), and molecular function (MF) results in Gene Ontology (GO) analysis. Kyoto Encyclopedia of Genes and Genomes (KEGG) pathways were also predicted.

All fecal samples were transported to Benagen Technology (Wuhan, China). After extracting and quantifying the total DNA of samples, specific primers with barcodes were synthesized according to the full-length primer sequence, and the rRNA gene variable regions (single or consecutive multiple) or specific gene fragments were amplified by polymerase chain reaction. The products were purified, quantified, and homogenized to form a sequencing library. Qualified libraries were sequenced using the Illumina platform. By filtering and denoising the paired reads, the species composition of the samples was compared. Further analysis including alpha-diversity (ACE and Shannon indices), beta-diversity [non-metric multi-dimensional scaling (NMDS), analysis of similarities (ANOSIM)], linear discriminant analysis (LDA) effect size (LEfSe), and KEGG metabolic pathways were used to explore the differences between samples.

### 2.4. Serum Biochemical Analysis

ELISA was performed per the manufacturer’s recommendations. Serum levels of alkaline phosphatase (ALP), alanine aminotransferase (ALT), aspartate aminotransferase (AST), total cholesterol (TC), triglyceride (TG), and low-density lipoprotein cholesterol (LDL-C) were determined by Servicebio (Wuhan, China). Glutamyl cysteingl glycine (GSH), catalase (CAT), MDA, superoxide dismutase (SOD), interleukin-6 (IL-6), IL-1β, and tumor necrosis factor-alpha (TNF-α) activities in liver tissues were measured using assay kits (Solarbio, Beijing, China).

### 2.5. Tissue Staining and Immunohistochemistry

Mouse liver sections (5 μm thick, formalin-fixed and paraffin-embedded) were prepared and stained with hematoxylin and eosin (H&E, Servicebio, Wuhan, China). The frozen cryosectioned liver samples were stained with oil red O to detect the lipid content (Servicebio). Liver histomorphology was assessed under a microscope. Anti-4-HNE antibody (K009925P, 1:500, Solarbio, Beijing, China) was used to perform immunohistochemistry.

### 2.6. Western Blotting

Total protein was extracted from mouse liver and ileum samples, quantified by the bicinchoninic acid (BCA) method, separated by 10% SDS-PAGE, and transferred onto polyvinylidene fluoride membranes. The membranes were blocked for 30 min with 5% (*w*/*v*) skimmed milk and incubated overnight with primary antibodies at 4 °C. Primary antibodies against histone H3 (GB11102), nuclear transcription factor p65 (GB11997), toll-like receptor (TLR4, GB11519), cluster of differentiation 14 (CD14, GB11254), inhibitor of nuclear factor kappa-B (IκBα, GB111509), Occludin (GB11149), clostridium perfringens enterotoxin receptor 4 (Claudin-4, GB114056), and glyceraldehyde-3-phosphate dehydrogenase (GAPDH, GB15002) were purchased from Servicebio. Primary antibodies against myeloid differentiation primary response protein (MyD88, 23230-1-AP) and zonula occludens (ZO)-1 (21773-1-AP) were obtained from the Proteintech Group (Wuhan, China). Primary antibodies were used at a dilution of 1:1000 (1:2000 for GAPDH). After washing with Tween (20)-tris salt buffer solution, the membranes were incubated with secondary antibodies (1:5000, 30 min, 25 °C). Electrochemiluminescence chemiluminescence and the AIWBwell™ imaging system (Servicebio) were used to collect quantified images.

### 2.7. Statistical Analysis

All statistical analyses were performed using the Paired Comparison Plot App (Origin 2021). Data were expressed as the mean ± standard deviation. Differences between two groups were assessed for statistical significance using the two-tailed Student’s *t*-test, whereas a one-way ANOVA was used for multigroup comparisons. *p* ≤ 0.05, 0.01, 0.001 indicates for difference, significant difference, and extremely significant difference in results, respectively.

## 3. Results and Discussion

### 3.1. Component Analysis of PPC and Liver Transcriptomics Results in Mice with ALD

**Figure 1:**
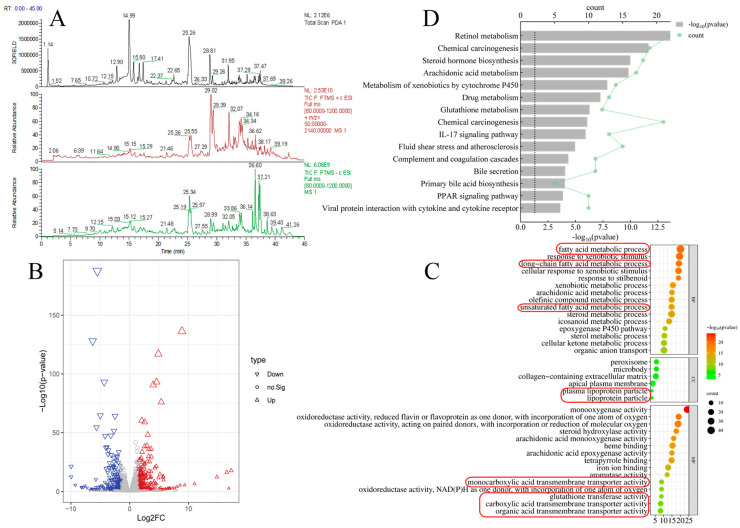
Component analysis of PPC and liver transcriptomics results of mice with ALD. (**A**) UPLC-MS/MS results of PPC. Black, red, and green lines represent for the UPLC (PDA detector), positive ion mode, and negative ion mode results, respectively. (**B**) Volcano map to screen for gene expression related to ALD using the screening criteria of |log_2_ (fold change)| > 1.5 and *p* < 0.05. The blue inverted and red positive triangle represent for the downregulated and upregulated genes. (**C**,**D**) GO (BP, MF, CC) and KEGG results for the selected genes related to ALD. GO annotations related to lipid metabolism are circled in red.

First, PPC was analyzed by UPLC-MS/MS to determine the components of the two species (Figure 1A, Supplementary Figure S1, and Table 1). Within 45 min, 10 compounds were identified, namely 3′-hydroxypuerarin (12.90 min), puerarin (14.99 min), 3′-methoxypuerarin (15.80 min), puerarin apioside (16.77 min), daidzin (17.41 min), daidzein (25.07 min), glycitein (25.57 min), luteolin (29.02 min), ursolic acid (36.51 min), and palmitic acid (37.43 min), in line with previous reports [8,12]. The anti-ALD activities of some of the aforementioned compounds have been reported in the literature [5,10], and they will not be further elaborated. Organic acids and catechin derivatives [13] in PPC were not detected well in this study because the high polarity of organic acids and catechin derivatives led to rapid retention times on the C18 column (1.14 min in Figure 1A, PDA detector results), and these compounds could not be separated from the baseline. In general, PPC has a substantial basis for alleviating ALD.

Next, liver transcriptomic sequencing results of mice from a public database (GSE 179398) [14] were analyzed to predict genes significantly associated with ALD. In total, 15,329 candidate genes were used to screen for associations with ALD (Figure 1B) using the criteria of |log2 (fold change)| > 1.5 and *p* < 0.05. Of these, 520 genes (297 upregulated and 223 downregulated genes) were selected for GO analysis (including BP, CC, and MF) to predict possible KEGG pathways (Figure 1C,D). The GO results suggested that genes related to lipid metabolism were important in the early stages of ALD in mice for multiple reasons. First, the metabolic processes of fatty acids, long-chain fatty acids, and unsaturated fatty acids were ranked in the top 15 important events of BP [in descending order by −log_10_(*p*-value), the same standard for BP, CC, and MF]. Second, the CC results illustrated that the location of those events might be plasma lipoprotein or lipoprotein particles. Third, after the occurrence of ALD, the activities of the monocarboxylic acid transporter, glutathione transfer, carboxylic acid transport transporter, and organic acid transporter are liable to change, and these functions were well matched with the bioactivities of the flavonoids, triterpenes, and potential organic acids in PPC. Together with the KEGG results (steroid hormone biosynthesis, arachidonic acid metabolism, and primary bile acid biosynthesis can be attributed to lipid metabolism subcategory), lipid metabolism dysfunction can be regarded as a landmark event in the early stages of ALD.

### 3.2. 16S rRNA Results of Mice with ALD with/without PPC Administration

**Figure 2:**
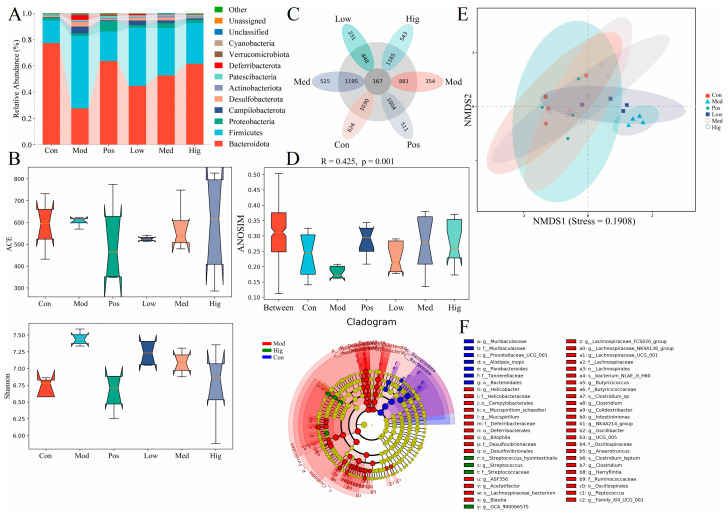
16S rRNA results of mice with ALD with/without PPC administration (*n* = 4). (**A**) Bar chart of species composition at the phylum level. (**B**) Differences in alpha-diversity (ACE and Shannon indices). (**C**) Petal diagram with common unique features. (**D**) ANOSIM based on the weighted Unifrac algorithm (R = 0.425, *p* = 0.001). (**E**) NMDS analysis based on the weighted Unifrac algorithm (Stress = 0.1908). (**F**) Cladogram of LEfSe. o, order; f, family; g, genus; s, species. Con, control group; Mod, Baijiu treatment group; Pos, silybin group; Low, low PPC dosing group; Med, medium PPC dosing group; Hig, high PPC dosing group.

To further investigate the effect of PPC in alleviating ALD in mice, we collected mouse feces for 16S rRNA sequencing. Figure 2A,C presents the distribution of gut microbes in mice with ALD (Figure 2A) and common species (167 species, Figure 2C). In healthy mice, *Bacteroidota* accounted for the majority of intestinal microbiota. In the Mod group, the abundance of *Bacteroidota* was reduced, and that of *Firmicutes* was significantly increased, in line with the reference data reported in non-alcoholic liver disease [15]. Alpha-diversity reflects the species richness and diversity of individual samples, with the ACE index measuring species abundance and the Shannon index reflecting species diversity. Under the same species abundance, an increase in the evenness of each species in a community results in an increase in the diversity of the community, resulting in the larger Shannon index. Alcohol disrupted the diversity of the gut microbiota in mice with ALD (Figure 2B), and this effect was rescued by PPD and silymarin administration.

Beta-diversity is used to compare the degree of similarity in species diversity among different samples, as presented in Figure 2D,E. NMDS is a non-linear statistical analysis method used to overcome the inclusion of linear analysis in methods such as principal component analysis or principal coordinates analysis. It can better reflect the nonlinear structure of ecological data. The tight arrangement of each point in NMDS (Figure 2E) indicates that the improvement in liver function in mice with ALD in the Med, Hig, and Pos groups was closer to that of the Con group, whereas that in the Low group was closer to that of the Mod group. The stress of NMDS was lower than 0.2 (0.1908), suggesting that the group of samples had a certain degree of reliability. ANOSIM (Figure 2D) is a nonparametric test used to assess intergroup and intragroup differences to determine whether the grouping is meaningful. The R value from the six groups of mice was 0.425, indicating that the samples from each group of mice had certain differences (R = 1 means the intergroup difference is greater than the intragroup difference), and such results have high reliability (*p* < 0.05). LEfSe can help identify biomarkers with statistical differences between different groups [16]. The distribution bar chart of LDA values (Supplementary Figure S2) and the evolutionary branching chart of LEfSe were used to identify biomarkers with significant differences among the Con, Mod, and Hig groups (Figure 2F). Based on the characteristics of the branching chart, gut microbial communities representing the three groups were identified (the sequence was based on the larger classification level, i.e., order, family, genus and species), namely *Bacteiroidales* for the Con group; *Bilophila*, *Campylobacterales*, *Deferribacterales*, *Desulfovibrionales*, *Lachnospirales*, and *Oscillospirales* for the Mod group, and *Streptococcus* for the Hig group.

**Figure 3:**
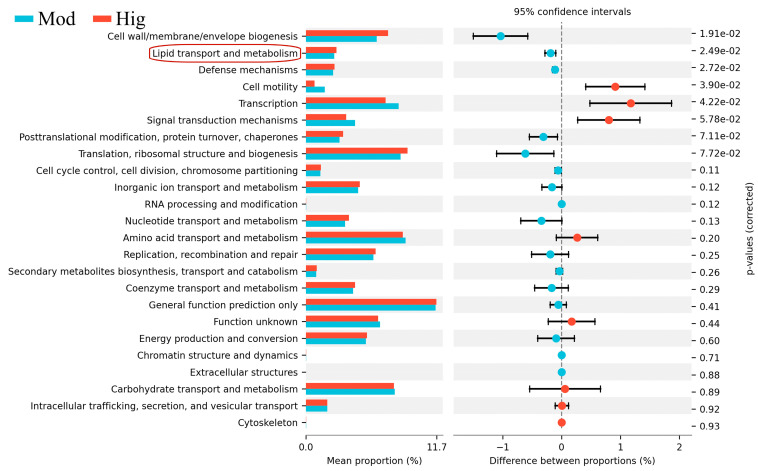
KEGG results of 16S rRNA in mice with ALD with/without PPC administration. Mod, Baijiu treatment; Hig, high dose of PPC. An annotation related to lipid metabolism is circled in red. 7.72e-2 = 0.0772, the same representation for the remaining data.

Lastly, KEGG functional differences were analyzed to summarize the changes in the functional genes of gut microbial communities involved in metabolic pathways among samples from the Mod and Hig groups (Figure 3). At a 95% confidence interval and *p* < 0.05, PPC administration enhanced the function of lipid transport and metabolism in mice with ALD. This result echoed the finding that lipid metabolism dysfunction is a characteristic phenotype of ALD in liver transcriptomic results, indicating that PPC can effectively alleviate ALD.

### 3.3. Validation of Liver Transcriptomics and Gut Microbiota Results

**Figure 4:**
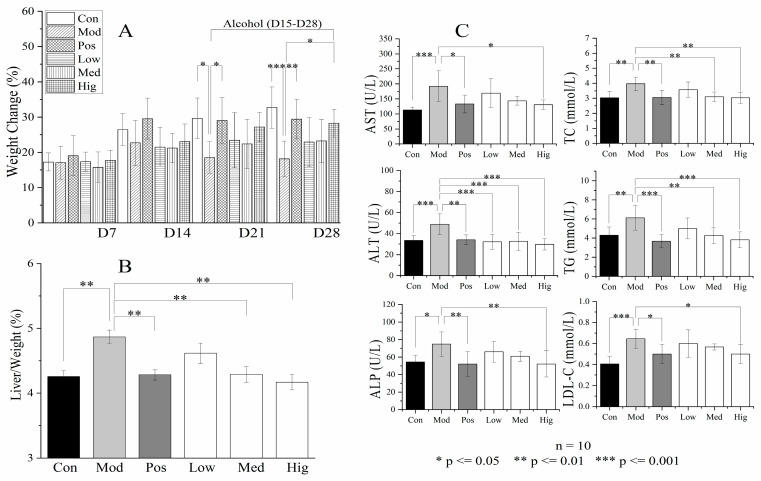
(**A**) Weight gain, (**B**) liver index, and (**C**) serum biochemical indicators of liver function (AST, ALT, ALP, TC, TG, LDL-C) in mice with ALD in the different groups (*n* = 10). ELISA results were displayed as enzyme activity concentration (U/L) or molar concentration (mmol/L). Statistical comparisons were made between the control group and the Baijiu group, as well as between the Baijiu group and the treatment group. Data were expressed as the mean ± standard deviation. *p* ≤ 0.05, 0.01, 0.001 indicates for difference, significant difference, and extremely significant difference in results, respectively. Con, control group; Mod, Baijiu treatment group; Pos, silymarin group; Low, low-dose PPC group; Med, medium-dose PPC group; Hig, high-dose PPC group.

**Figure 5:**
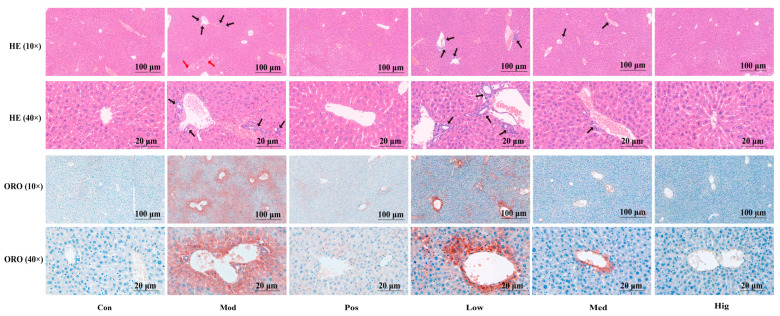
H&E (HE) and oil red O (ORO) staining of liver tissue. In the histological images, the magnification of the microscope is 10× and 40×, and the length of the scale bar is 100 μm and 20 μm. Black arrow, location of lymphocyte infiltration, red arrow, degenerated and necrotic liver cells. Con, control group; Mod, Baijiu treatment group; Pos, silymarin group; Low, low-dose PPC group; Med, medium-dose PPC group; Hig, high-dose PPC group.

We designed animal experiments to verify the liver transcriptomics and gut microbiota results. Figure 4A presents the rapid increment of weight (gain value/original value, *g*/*g*) in mice with ALD (Mod) at week 3 (days 15–21), and the results were significant at week 4 (days 22–28). Compared with the effects of silymarin, high-dose PPC suppressed the weight change over days 22–28 with no obvious liver lesions (Supplementary Figure S3), together with the normal bright red liver color. The H&E staining results (Figure 5, HE) suggested that alcohol induced an irregular arrangement of hepatocytes, lipid vesicles in cytosolic compartment and inflammatory infiltration in hepatocytes (Mod, Low group); these changes were relieved by the treatments with high-dose PPC or silymarin. The results of oil Red O (ORO) showed that compared with the Con group, the Mod and Low group exhibited severe droplets of neutral lipid, while such a situation was alleviated in the Pos, Med, and Hig groups (Figure 5, ORO). High-dose PPC also decreased the accumulation of the end-product of lipid peroxidation 4-HNE from the immunohistochemistry results (Supplementary Figure S4). The results for liver indices and functional biochemical indicators (AST, ALT, ALP, TC, TG, LDL-C) revealed that Baijiu significantly increased the organ index and the levels of the corresponding biochemical indicators, and these changes were reversed by high-dose PPC (Figure 4B,C).

**Figure 6:**
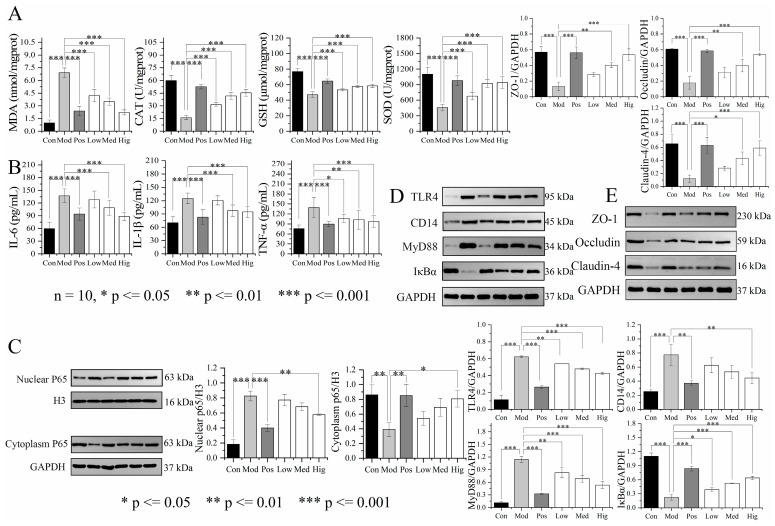
PPC alleviates inflammation in mice with ALD through antioxidant activity and the restoration of intestinal barrier integrity (*n* = 10). (**A**,**B**) ELISA results of serum antioxidant stress and inflammatory markers (MDA, CAT, GSH, SOD, IL-6, IL-1β and TNF-α), displayed as mass (pg), molar (μmol, nmol) or enzyme activity units (U) of the detection marker per volume units (mL) or mgprot. (**C**–**E**) WB results of NF-κB pathway (TLR4, CD14, IκBα, and P65) and intestinal proteins (ZO-1, occludin and claudin-4). Statistical comparisons were made between the control group and the Baijiu model group, as well as between the Baijiu model group and the treatment group. Data were expressed as the mean ± standard deviation. *p* ≤ 0.05, 0.01, 0.001 indicates for difference, significant difference, and extremely significant difference in results, respectively. Con, control group; Mod, Baijiu treatment group; Pos, silymarin group; Low, low-dose PPC group; Med, medium-dose PPC group; Hig, high-dose PPC group.

In addition, the anti-oxidative stress and anti-inflammatory effects of PPC in mice with ALD were studied. The results of ELISA revealed significant decreases in CAT GSH, and SOD concentrations in mice with ALD compared with the findings in the Con group. After PPC administration, the levels of markers increased to normal rates, especially in the Hig group, similar to those observed in the Pos group (Figure 6A). MDA is a product of lipid peroxidation that can modify DNA bases to form carcinogenic exocyclic etheno–DNA adducts [17]. Mice with ALD experienced a significant increase in the MDA concentration, causing the release of inflammatory factors (IL-6, IL-1β, and TNF-α). These indicators returned to normal levels after PPC administration, and the extent of recovery in the Hig group approximated to that in the Pos group (Figure 6B). Nuclear factor kappa-B (NF-κB) is a key transcription factor that regulates the expression of pro-inflammatory genes. Exposure to pro-inflammatory factors such as TNF-α induces IκBα phosphorylation, leading to NF-κB activation and its nuclear translocation as well as the production of a large number of inflammatory factors [18]. As presented in Figure 6C,D, compared with the findings in the Con group, the protein expression of nuclear factor p65 was significantly increased in the Mod group, with Baijiu exposure leading to high expression of TLR4, CD14, MyD88, and cytoplasmic P65 and the inhibition of IκBα (*p* < 0.001). PPC inhibited TLR4, CD14, and MyD88 expression in the liver tissue of mice with ALD and enhanced the protein expression of IκBα, resulting in regulation of the NF-κB pathway, inhibition of inflammatory factors and liver inflammation levels, and the restoration of liver function. Occludin, claudin-4, and ZO-1 are tight junction proteins expressed on the intestinal mucosa that protect the intestinal mucosal barrier from exogenous damage [19]. As illustrated in Figure 6E, compared with the findings in the Con group, occludin, claudin-4, and ZO-1 expression was significantly decreased in the ileal tissue of mice in the Mod group (*p* < 0.001), indicating impairment of the intestinal barrier in mice with ALD and the occurrence of an inflammatory response. The administration of silymarin or high-dose PPC significantly increased the protein expression of occludin, claudin-4, and ZO-1, thereby maintaining the integrity of the intestinal barrier (*p* < 0.001).

### 3.4. Discussion

*P. lobata* and *P. mume* are common plants with medicinal and food functions. According to literature reports, starch, fermentation, or processed products from the roots of *P. lobata* and the fruits of *P. mume* can exhibit a regulation effect on the gut microbiota [15,20,21,22]. The greatest significance between PPC and previous reports is the influenced species in gut microbiota. From the results of 16S rRNA sequencing, the abundance of *Muribaculaceae* and *Bacteroides* (at the genus level), which can inhibit inflammation and improve intestinal barrier function [23], was decreased by alcohol exposure. Together with *Streptococcus*, the predominant bacterium used to predict the severity of liver injury in ALD [24], the abundance of these microbes was restored by PPC administration (Figure 2F, Supplementary Figure S2). These results are different from *Lactobacillus,* which was recorded by previous data [20,21,22]. The reason may be related to the compounds in PPC. The LC-MS results displayed that PPC contained flavonoids and triterpenoids such as puerarin, ursolic acid and their derivatives. Isoflavonoids from the roots of *P. lobata*, phenolic acids, and triterpenoids from the fruits of *P. mume* have exhibited antioxidant activity in vitro and could be used as nonenzymatic antioxidants [25]. These compounds can enhance the activity of oxygen-scavenging enzymes (including SOD, GSH-PX, and CAT). In addition, flavonoids and triterpenoids can exert anti-inflammatory effects and maintain intestinal barrier integrity via TLR4-NF-κB signaling pathway and tight junction proteins (ZO-1, claudins, and occludin) [26].

To avoid repetition, we did not design antioxidant experiments in vitro and it may be one weakness of this work. Meanwhile, the phenolic acids in PPC generated a mixed chromatographic peak at the initial stage of LC-MS detection (retention time of 1.14 min) because of their high polarity, representing a limitation of the results in this study. By contrast, the dosage in the high-dose PPC group was 146.34 mg/kg, which was lower than the dose of 210 mg/kg or 400 mg/kg reported previously [5,15]. Thus, PPC has development and application advantages in the future.

## 4. Conclusions

We conducted in-depth research on the hepatoprotective effects of PPC against ALD in mice and validated the mechanisms. Liver transcriptomics and 16S rRNA sequencing suggested that PPC corrects lipid metabolism dysfunction in the early stages of ALD. This was reflected by reduced alcohol-induced hepatomegaly and lipid accumulation; the normalization of serum AST, ALT, ALP, TC, TG, and LDL-C levels, and decreases in the MDA concentration. These changes enhanced anti-oxidative stress responses by improving SOD, GSH, and CAT activities and preventing the release of the inflammatory factors IL-6, IL-1β, and TNF-α. PPC can regulate the NF-κB signaling pathway by downregulating TLR4, CD14, and MyD88 proteins, thereby inhibiting the release of inflammatory factors, and restoring the expression of occludin, claudin-4, and ZO-1, thus maintaining the integrity of the intestinal mucosal barrier. Moreover, PPC displayed a certain restorative effect on the dysbiosis of the gut microbiota in mice with ALD. Although the antioxidant activity of the extract was not further tested in vitro, the dosage of PPC that effectively alleviated ALD in this study was lower than that reported previously. In general, our results provide a basis for the subsequent development and application for other functional foods or drugs containing *P. lobata* and *P. mume* to alleviate ALD.

## Figures and Tables

**Table 1:** UPLC-MS/MS results of the main compounds in PPC.

No	Retention Time (min)	Compound Name	Molecular Weight	Adduct Ion	Molecular Formula	MS/MS Fragments
1	12.90	3′-Hydroxypuerarin	431.0995	[M−H]^−^	C_21_H_20_O_10_	431.0991, 311.0566, 283.0616
2	14.99	Puerarin	415.1047	[M−H]^−^	C_21_H_20_O_9_	415.1043, 295.0615, 267.0677
3	15.80	3′-Methoxypuerarin	445.1153	[M−H]^−^	C_22_H_22_O_10_	445.1148, 325.0723, 282.0539
4	16.77	Puerarin apioside	547.1473	[M−H]^−^	C_26_H_28_O_13_	547.1467, 295.0616, 267.0667
5	17.41	Daidzin	417.1183	[M+H]^+^	C_21_H_20_O_9_	297.0748, 255.0656, 199.0754
6	25.07	Daidzein	253.0512	[M−H]^−^	C_15_H_10_O_4_	253.0509, 209.0609, 135.0078
7	25.57	Glycitein	283.0621	[M−H]^+^	C_16_H_12_O_5_	283.0616, 268.0381, 211.0394
8	29.02	Luteolin	285.0414	[M−H]^−^	C_15_H_10_O_6_	285.0409, 255.0300, 151.0031
9	36.51	Ursolic acid	455.3546	[M−H]^−^	C_30_H_48_O_3_	455.3540, 407.3370, 238.0641
10	37.43	Palmitic acid	255.2334	[M−H]^−^	C_16_H_32_O_2_	255.2332, 233.1788, 193.8156

## Data Availability

The original contributions presented in the study are included in the article/Supplementary Materials, further inquiries can be directed to the corresponding authors.

## Supplementary Materials

The following supporting information can be downloaded at https://www.mdpi.com/article/10.3390/foods13152431/s1: Figure S1. MS/MS of the main compounds from the *Pueraria lobata*–*Prunus mume* complex; Figure S2. Bar chart of linear discriminant analysis effect size; Figure S3. Liver size of mice with ALD with/without drug administration; Figure S4. Immunohistochemistry results (4-HNE) of the liver of mice with ALD (×40).
