# Supplementary material for: Pueraria lobata–Prunus mume Complex Alleviates Alcoholic Liver Disease by Regulating Lipid Metabolism and Inhibiting Inflammation: A Transcriptome and Gut Microbiota Analysis
Source: Foods. 2024 Aug 1;13(15):2431. doi: 10.3390/foods13152431 (PMC11311716; doi:10.3390/foods13152431)
Supplement: Supplementary file 1 [file foods-13-02431-s001.zip › foods-3082887-supplementary.pdf]

Supplementary material

Pueraria lobata–Prunus mume Complex Alleviate Alcoholic Liver Disease by Regulating Lipid Metabolism and Inhibiting Inflammation: A Transcriptome and Gut Microbiota Analysis

Ruixi Gao [1], Qi Huang [1], Yanfeng Zeng [1], Dandan Chen [1], Ziming Jia [2,*], Bingchen Han [3], Xianju Huang [1], Qiang Wang [1], Xin Hu [1], Maochuan Liao [1], and Jun Li [1,4,*]

1 School of Pharmaceutical Sciences, South-Central Minzu University, Wuhan 430074, China;
2 Center for Disease Control and Prevention, Hubei Provincial, Wuhan 430079, China;
3 College of Life Sciences, South-Central Minzu University, Wuhan 430074, China
4 International Science and Technology Cooperation Base for Evaluation and Utilization of Traditional Medical Resources, South-Central Minzu University, Wuhan 430074, China
* Correspondence: jiaziming2024@163.com (Z. J.), lijun-pharm@hotmail.com (J. L.).

Contents

Supplementary Figure:

S1	MS/MS of the main comounds from *Pueraria lobata* and fruit of *Prunus mume* complex.	PP. 3-4
S2	Bar chart of line discriminant analysis (LDA) effect size.	PP. 5
S3	Liver size of ALD mice with/without drug administration.	PP. 6
S4	Immunohistochemistry results (4-HNE) of ALD mice liver (40x).	PP. 7

Compound 1

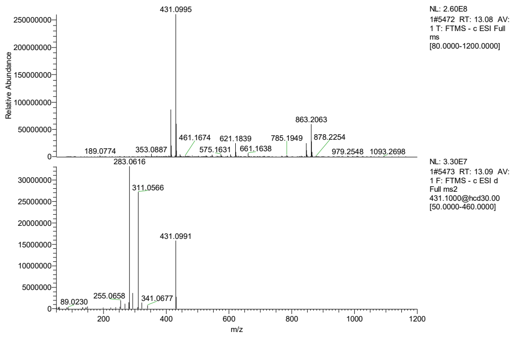

Compound 2

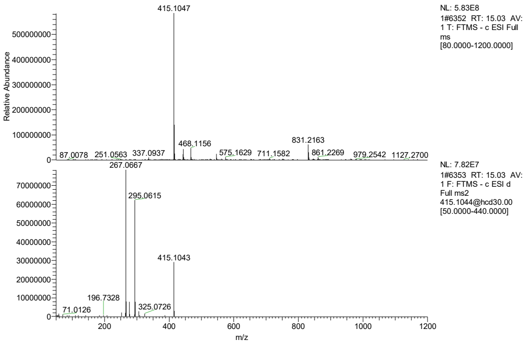

Compound 3

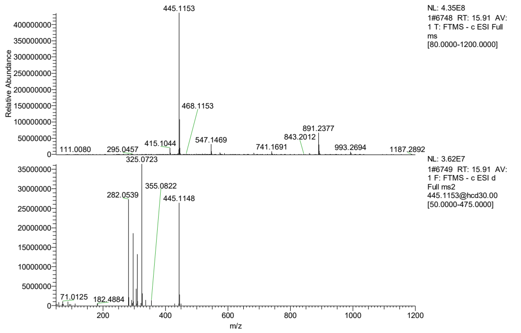

Compound 4

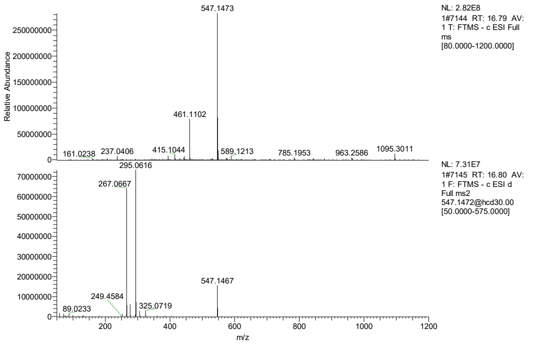

Compound 5

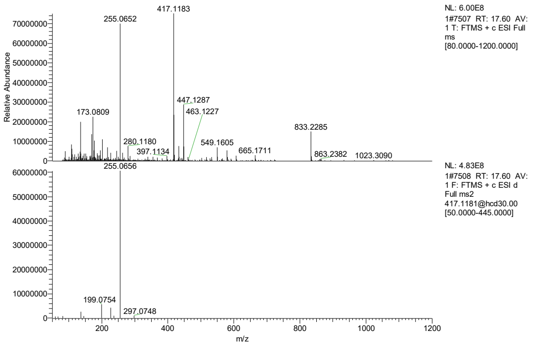

Compound 6

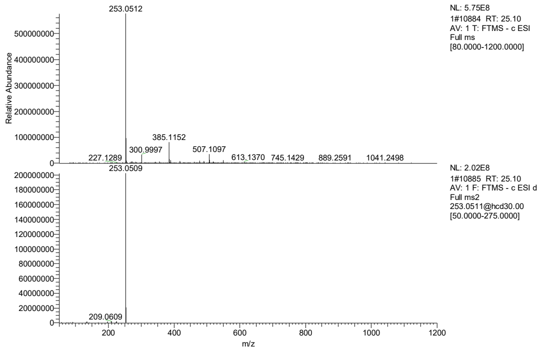

Compound 7

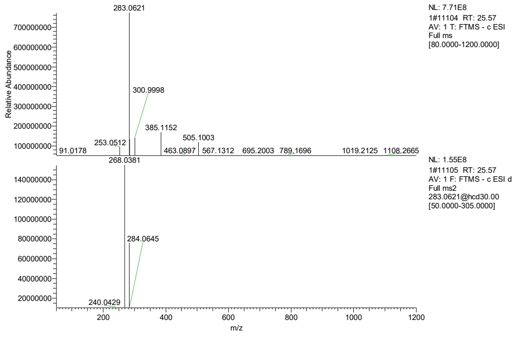

Compound 8

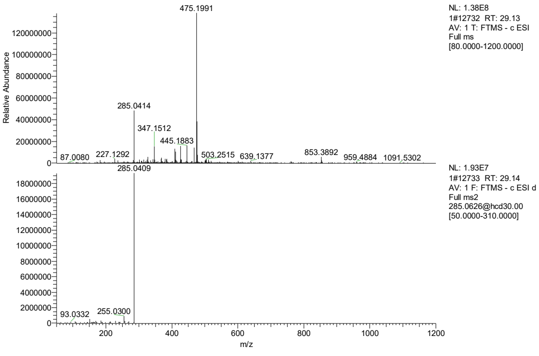

Compound 9

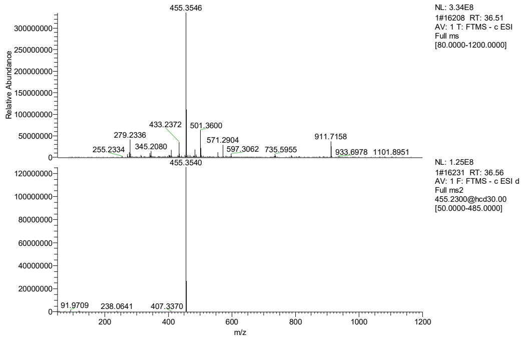

Compound 10

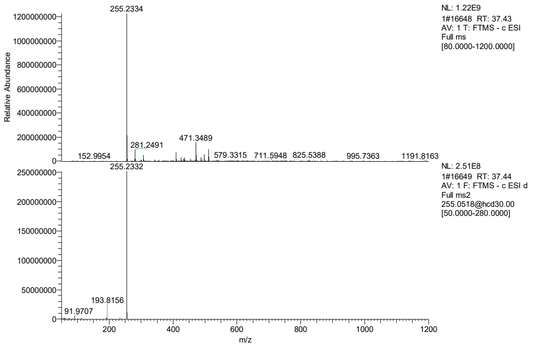

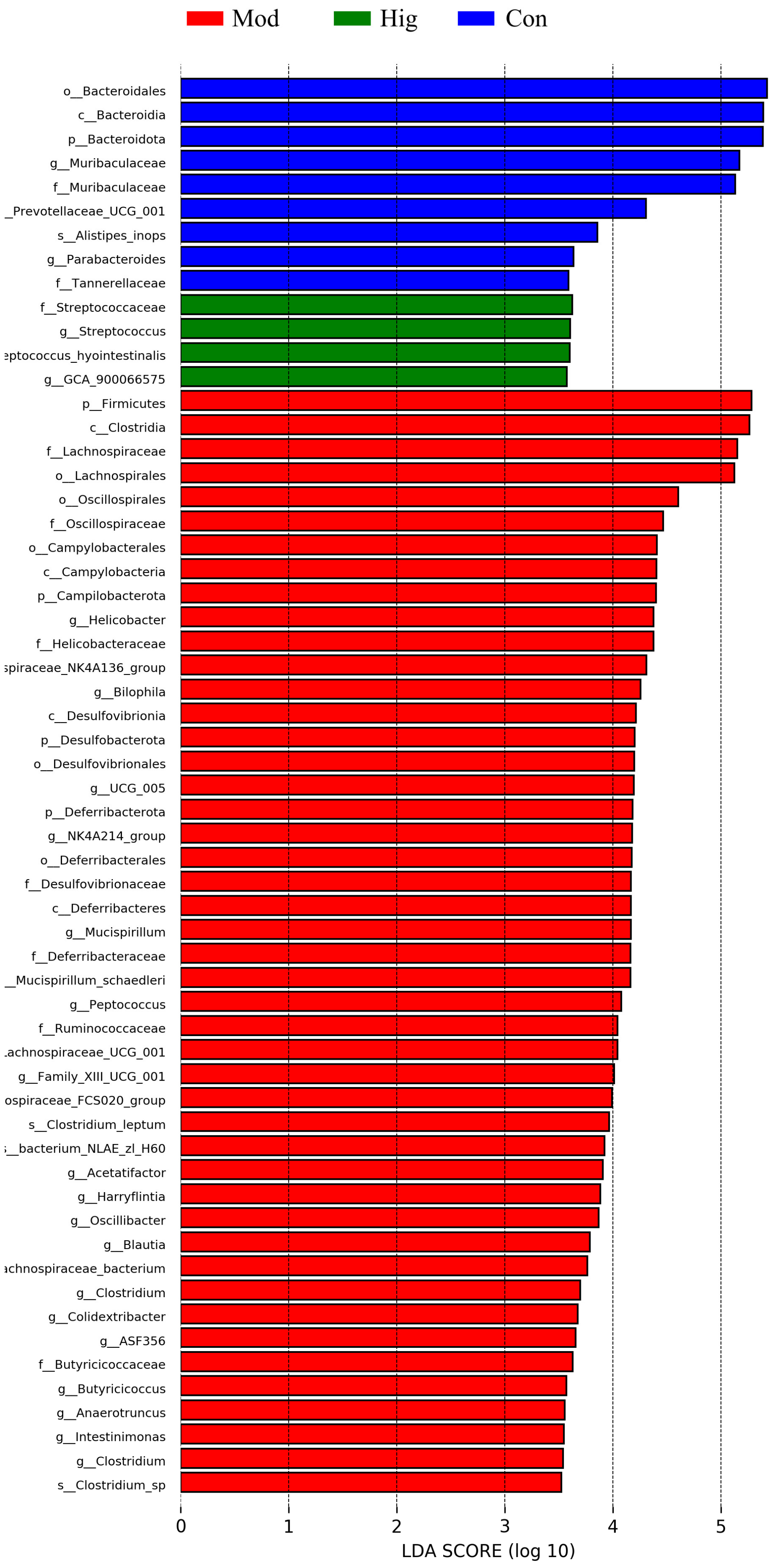

Mod
Hig
Con
o__Bacteroidales
c__Bacteroidia
p__Bacteroidota
g__Muribaculaceae
f__Muribaculaceae
_Prevotellaceae_UCG_001
s__Alistipes_inops
g__Parabacteroides
f__Tannerellaceae
f__Streptococcaceae
g__Streptococcus
eptococcus_hyointestinalis
g__GCA_900066575
p__Firmicutes
c__Clostridia
f__Lachnospiraceae
o__Lachnospirales
o__Oscillospirales
f__Oscillospiraceae
o__Campylobacterales
c__Campylobacteria
p__Campilobacterota
g__Helicobacter
f__Helicobacteraceae
spiraceae_NK4A136_group
g__Bilophila
c__Desulfovibrionia
p__Desulfobacterota
o__Desulfovibrionales
g__UCG_005
p__Deferribacterota
g__NK4A214_group
o__Deferribacterales
f__Desulfovibrionaceae
c__Deferribacteres
g__Mucispirillum
f__Deferribacteraceae
__Mucispirillum_schaedleri
g__Peptococcus
f__Ruminococcaceae
achnospiraceae_UCG_001
g__Family_XIII_UCG_001
ospiraceae_FCS020_group
s__Clostridium_leptum
__bacterium_NLAE_zl_H60
g__Acetatifactor
g__Harryflintia
g__Oscillibacter
g__Blautia
achnospiraceae_bacterium
g__Clostridium
g__Colidextribacter
g__ASF356
f__Butyricicoccaceae
g__Butyricicoccus
g__Anaerotruncus
g__Intestinimonas
g__Clostridium
s__Clostridium_sp
0
1
2
3
4
5
LDA SCORE (log 10)

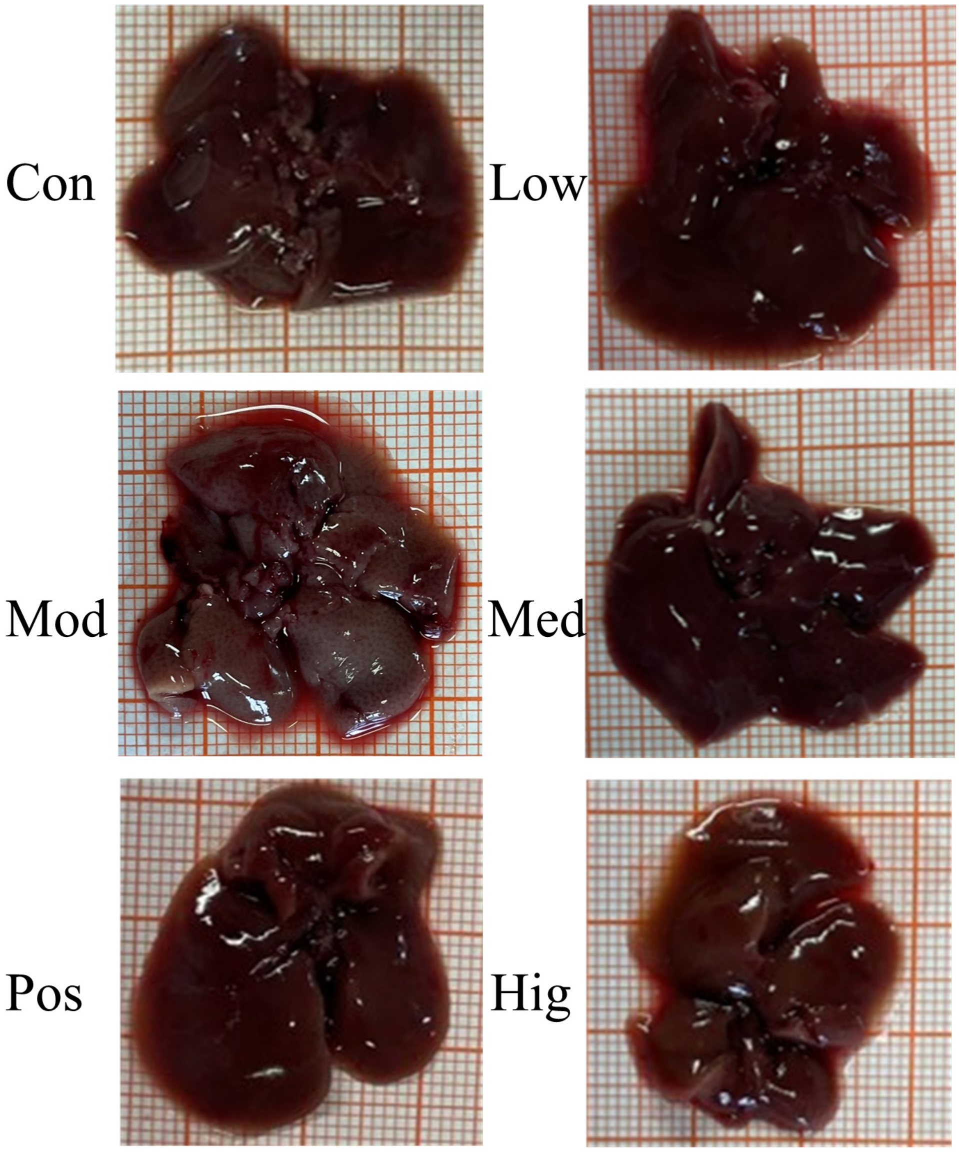
Con
Low
Mod
Med
Pos
Hig

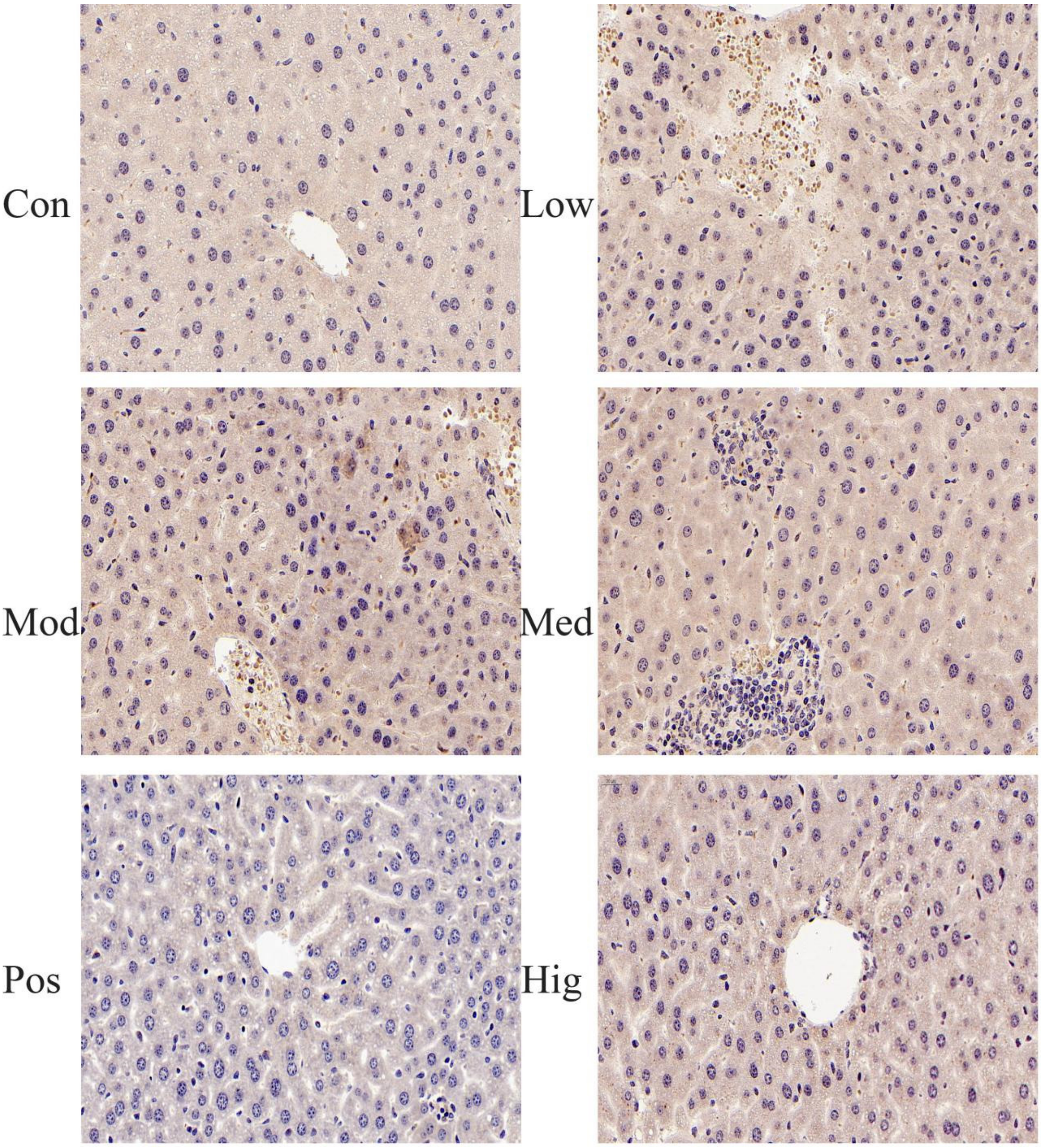
Con
Low
Mod
Med
Pos
Hig
